# Grade-Dependent Prognostic Value of Classical Clinicopathological Factors in Glioma: A Single-Center Retrospective Study

**DOI:** 10.3390/jcm15114372

**Published:** 2026-06-05

**Authors:** Ge Zhang, Mengting Zhang, Xuetao Han, Lixia Zhou, Liubing Hou, Yanqiang Wang, Xiaoying Xue, Huandi Zhou

**Affiliations:** 1 Department of Radiotherapy, The Second Hospital of Hebei Medical University, Shijiazhuang 050061, China; gezhang@hebmu.edu.cn (G.Z.);; 2 Department of the Third Oncology, Handan Central Hospital, Handan 056001, China; 3 Department of Radiology, The Second Hospital of Hebei Medical University, Shijiazhuang 050061, China

**Keywords:** glioma, prognosis, WHO grade, anatomical location, Ki-67

## Abstract

**Background:** The prognostic relevance of classical clinicopathological factors in glioma may vary across World Health Organization (WHO) grades. We examined whether anatomical location, Ki-67, and treatment-related variables show grade-dependent associations with survival. **Methods:** We retrospectively included 429 patients with glioma treated at a single center between 2012 and 2024. Patients were stratified into WHO grade 2–3 gliomas (*n* = 129) and WHO grade 4 gliomas (*n* = 300). Overall survival was evaluated using Kaplan–Meier analysis and Cox proportional hazards models. Additional exploratory analyses were performed in cases with known IDH status with adjustment for IDH and MGMT status. **Results:** Median overall survival for the full cohort was 29.93 months, and survival differed significantly across WHO grades. In grade 2–3 gliomas, higher Karnofsky Performance Status, non-deep location, and lower Ki-67 were associated with better survival; deep/midline location and Ki-67 ≥ 30% remained independently associated with worse overall survival. Within non-deep grade 2–3 tumors, temporal location was associated with worse survival. In grade 4 gliomas, female sex, non-deep location, and receipt of radiotherapy were independently associated with better survival, whereas Ki-67 was not prognostic. In exploratory molecularly adjusted analyses, deep/midline location remained associated with worse survival across grades, whereas the prognostic effect of Ki-67 became unstable. Longer chemotherapy duration showed a trend toward improved survival in grade 4 gliomas. **Conclusions:** Classical clinicopathological factors show substantial grade-dependent prognostic heterogeneity in glioma. Anatomical location appears to be a relatively stable adverse prognostic factor across grades, including after partial molecular adjustment. In contrast, the prognostic relevance of Ki-67 was less robust after adjustment for IDH and MGMT and should be interpreted cautiously as an exploratory, molecular-context-dependent marker.

## 1. Introduction

Gliomas are among the most common primary tumors of the central nervous system and are characterized by substantial heterogeneity in histology, molecular features, clinical behavior, and survival [1,2]. The 2021 World Health Organization Classification of Tumors of the Central Nervous System has further emphasized the central role of molecular pathology in glioma classification, integrating key alterations such as isocitrate dehydrogenase mutation status and 1p/19q codeletion into the diagnostic framework [3,4]. However, despite this shift toward molecularly defined entities, conventional clinicopathological variables—including age, Karnofsky performance status, tumor grade, extent of resection, treatment exposure, anatomical location, and proliferative activity—remain highly relevant in routine clinical assessment and prognostic evaluation [5].

Importantly, the prognostic relevance of these classical factors may not be uniform across WHO grades. Lower-grade gliomas, including WHO grade 2 and grade 3 tumors, often show relatively prolonged and heterogeneous clinical courses, in which functional status, tumor location, extent of resection, and proliferative activity may provide meaningful survival stratification [6,7]. By contrast, WHO grade 4 gliomas, particularly glioblastomas, are characterized by highly aggressive biological behavior and short survival, and their outcomes are strongly influenced by treatment-related variables such as radiotherapy and temozolomide-based chemotherapy [8,9]. As a result, prognostic signals observed in one grade group may not be directly transferable to another.

Among conventional variables, anatomical location and Ki-67 are of particular interest. Tumors arising in deep, central, or eloquent brain regions may be associated with worse outcomes because of limited surgical accessibility, proximity to functionally important areas, and diffuse infiltrative growth patterns [10]. Ki-67 is a widely used marker of proliferative activity, but its prognostic value in glioma remains inconsistent across different pathological entities and grades [11,12,13]. In addition, treatment-related factors may carry distinct prognostic implications in high-grade disease, where therapeutic intensity and tolerability can substantially influence survival [8]. However, most existing studies have focused on single-grade populations, glioblastoma alone, or specific pathological subtypes, and direct comparisons of the prognostic roles of [8] classical clinicopathological factors across WHO grade groups remain limited. In particular, the grade-dependent effects of anatomical location, Ki-67, and treatment-related variables require further clarification.

In this study, we retrospectively analyzed a single-center cohort of patients with glioma and performed separate survival analyses for WHO grade 2–3 and WHO grade 4 tumors. We aimed to evaluate whether the prognostic value of classical clinicopathological variables differs by grade, with particular focus on anatomical location, Ki-67, and treatment-related factors. By identifying grade-dependent differences in prognostic factors, this study may contribute to more refined risk stratification and provide clinically relevant insights for individualized patient assessment in glioma.

## 2. Materials and Methods

### 2.1. Study Design and Patient Selection

This was a single-center retrospective cohort study. Consecutive patients with glioma who were diagnosed and treated at our institution between September 2012 and December 2024 were screened for eligibility. Eligible cases had histopathological confirmation following surgical resection or biopsy and had available clinical, radiological, pathological, and follow-up data. Tumors were classified according to the World Health Organization (WHO) classification of tumors of the central nervous system. For prognostic analyses, patients were stratified into WHO grade 2–3 gliomas and WHO grade 4 gliomas. Available pathological and molecular records were re-checked, and tumor classification was updated as much as possible according to the 2021 WHO framework. Cases lacking essential molecular information were not forcibly assigned to fully integrated molecular categories and were retained as NOS or classified according to the available diagnostic information.

Patients were included if they met all of the following criteria: (1) histopathologically confirmed glioma; (2) available clinical, pathological, and follow-up information; and (3) available imaging and anatomical location data for analysis. Patients were excluded if they had substantial missing clinical, pathological, or follow-up data; if tumor location, Ki-67, or treatment information could not be reliably determined; or if overall survival (OS) status was unavailable. A total of 429 patients were included in the final cohort, comprising 50 WHO grade 2 gliomas, 79 WHO grade 3 gliomas, and 300 WHO grade 4 gliomas.

### 2.2. Data Collection and Variable Definitions

Clinical and pathological data were collected from the electronic medical record system, radiology records, and pathology reports. Variables included sex, age, Karnofsky Performance Status (KPS), maximum tumor diameter, anatomical location, preoperative epilepsy, radiotherapy, chemotherapy, chemotherapy duration, and Ki-67 labeling index.

Tumor location was initially categorized as deep/midline or non-deep. Deep/midline tumors were defined as lesions involving deep or midline structures, including the thalamus, basal ganglia, corpus callosum, brainstem, or related central regions. All other lobar or more superficially located tumors were classified as non-deep. To further assess spatial heterogeneity within lower-grade tumors, non-deep WHO grade 2–3 gliomas were additionally categorized as temporal versus non-temporal.

Ki-67 was obtained from pathology reports and categorized into 3 groups: <20%, 20–30%, and ≥30%. Chemotherapy duration was defined according to the actual number of completed treatment cycles. In analyses examining chemotherapy duration, cases with unavailable cycle data were excluded. Sankey plots were used as descriptive visualizations to illustrate the distributions of anatomical location, Ki-67 strata, chemotherapy-duration strata, and OS categories.

### 2.3. Follow-Up and Outcome Definition

Survival data were obtained from outpatient follow-up records, inpatient records, and telephone follow-up. Follow-up was censored in September 2025. The primary endpoint was OS, defined as the time from the date of pathological diagnosis to death from any cause or the date of last follow-up. Patients who were alive at the last follow-up were censored.

### 2.4. Statistical Analysis

All statistical analyses and visualizations were performed using R software, version 4.2.1. Sankey plots were generated using the ggplot2 package, version 3.4.4, and the ggalluvial package, version 0.12.3. Survival analyses were performed using the survival package, version 3.3.1, the survminer package, version 0.4.9, and the rms package, version 6.3-0, as appropriate. Continuous variables were summarized as mean ± standard deviation or median with interquartile range, as appropriate, and categorical variables were summarized as counts and percentages.

OS was estimated using the Kaplan–Meier method and compared using the log-rank test. Cox proportional hazards regression models were used to evaluate associations between clinicopathological variables and OS. Univariable and multivariable analyses were performed separately for WHO grade 2–3 gliomas and WHO grade 4 gliomas. Variables showing statistical significance in univariable analysis were entered into multivariable Cox models. Hazard ratios (HRs) and 95% confidence intervals (CIs) were reported. The proportional hazards assumption was assessed using Schoenfeld residuals.

Prespecified subgroup analyses in WHO grade 2–3 gliomas included evaluation of deep/midline versus non-deep tumors, assessment of temporal versus non-temporal location within the non-deep subgroup, and analysis of the prognostic value of Ki-67 categories. In WHO grade 4 gliomas, additional analyses included evaluation of deep/midline versus non-deep tumors, Ki-67 categories, and chemotherapy duration. Analyses involving chemotherapy duration were restricted to patients with available cycle data and should be interpreted as exploratory. All tests were 2-sided, and *p* < 0.05 was considered statistically significant.

Additional exploratory Cox analyses were performed in cases with known IDH status, with adjustment for IDH and MGMT promoter methylation status, to assess the potential confounding effect of molecular classification. These analyses were considered exploratory because of incomplete molecular annotation.

### 2.5. Ethical Approval

This retrospective study was approved by the institutional ethics committee of our hospital. Given the retrospective design, the requirement for written informed consent was waived when applicable according to institutional policy.

## 3. Results

### 3.1. Patient Characteristics and Overall Survival in the Full Cohort

A total of 429 patients with glioma were included in this single-center retrospective cohort, all with complete clinicopathological and follow-up data. There was a male predominance in the overall cohort, consistent with previous epidemiological studies of glioma. The cohort comprised 50 WHO grade 2 gliomas, 79 WHO grade 3 gliomas, and 300 WHO grade 4 gliomas(Table 1). Given the predominance of grade 4 tumors and the apparent heterogeneity in clinical characteristics and treatment patterns across grades, subsequent analyses were performed in a grade-stratified manner.

**Figure 1:**
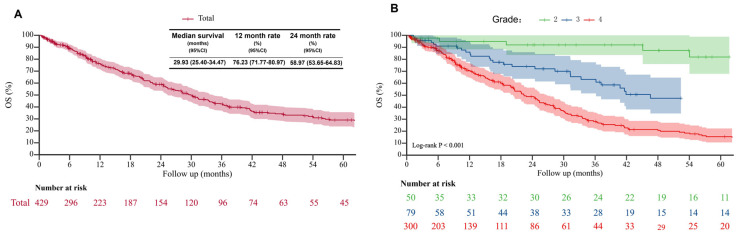
Overall survival in the full cohort (**A**) and by WHO grade (**B**).

At the time of follow-up censoring, the median overall survival (OS) of the entire cohort was 29.93 months. The 1-year and 2-year OS rates were 76.23% and 58.97%, respectively. Kaplan–Meier analysis demonstrated significant survival differences across WHO grades (log-rank *p* < 0.001), supporting separate prognostic analyses for grade 2–3 and grade 4 gliomas (Figure 1).

### 3.2. Prognostic Factors in WHO Grade 2–3 Gliomas

In patients with WHO grade 2–3 gliomas, univariable Cox regression showed that Karnofsky Performance Status (KPS), anatomical location, and Ki-67 were significantly associated with OS, whereas sex, age, tumor size, preoperative epilepsy, radiotherapy, and chemotherapy duration were not. Specifically, deep/midline tumors were associated with a significantly higher risk of death than non-deep tumors, and Ki-67 ≥ 30% was associated with worse survival compared with Ki-67 < 20%.

In multivariable analysis, KPS, anatomical location, and Ki-67 remained independently associated with OS. Higher KPS was associated with a lower hazard of death (HR 0.971, 95% CI 0.952–0.991, *p* = 0.005). Deep/midline location remained a strong adverse prognostic factor (HR 4.939, 95% CI 1.997–12.215, *p* < 0.001). In addition, Ki-67 ≥ 30% was independently associated with worse OS relative to Ki-67 < 20% (HR 2.194, 95% CI 1.064–4.526, *p* = 0.033), whereas Ki-67 20–30% was not significantly associated with survival (Table 2).

**Figure 2:**
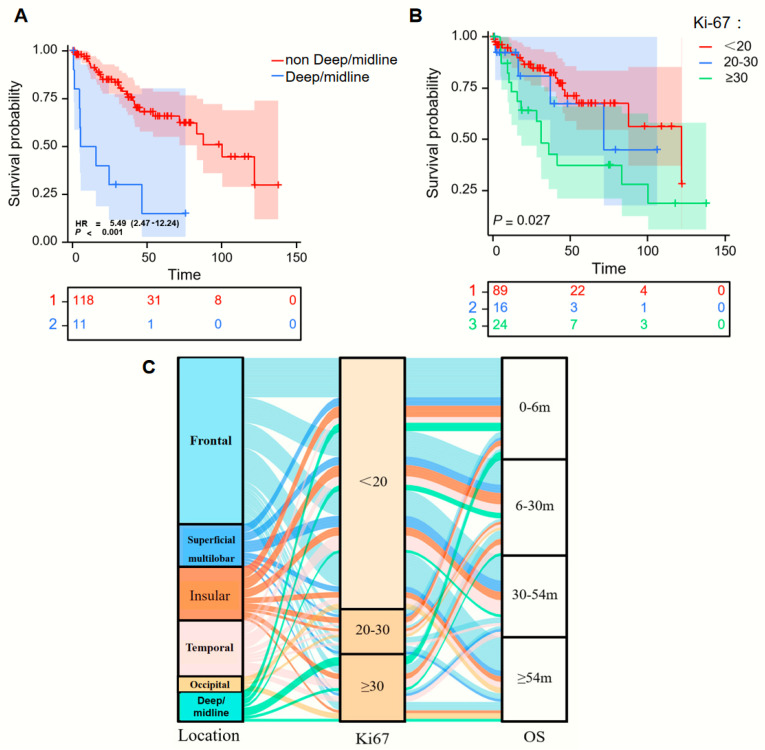
Survival differences according to anatomical location and Ki-67 expression in WHO grade 2–3 gliomas. (**A**) OS curves for deep/midline vs. non-deep/midline tumors. (**B**) OS curves stratified by Ki-67 expression categories. (**C**) Sankey diagram showing the combined effects of location and Ki-67 on survival outcomes.

Consistent with the regression findings, Kaplan–Meier analysis demonstrated that deep/midline tumor location was associated with significantly shorter overall survival compared with non-deep/midline location (log-rank *p* < 0.001, Figure 2A), and Ki-67 expression ≥ 30% conferred a worse prognosis relative to Ki-67 < 20% (log-rank *p* = 0.005, Figure 2B). Early separation of survival curves was observed in both comparisons, indicating that these prognostic differences emerged early in the disease course. To further visualize the combined prognostic impact of these two factors, we constructed a Sankey diagram (Figure 2C). This analysis revealed that deep/midline tumors were enriched for high Ki-67 expression (≥30%), and the combination of deep/midline location and Ki-67 ≥ 30% identified the subgroup with the poorest survival outcomes. Conversely, non-deep/midline tumors with Ki-67 < 20% had the most favorable prognosis.

However, in the exploratory analysis restricted to cases with known IDH status and adjusted for IDH and MGMT, the association between Ki-67 and OS was attenuated and no longer remained significant, whereas deep/midline location remained independently associated with worse OS (Supplementary Table S1).

### 3.3. Spatial Heterogeneity Within Non-Deep WHO Grade 2–3 Gliomas

**Figure 3:**
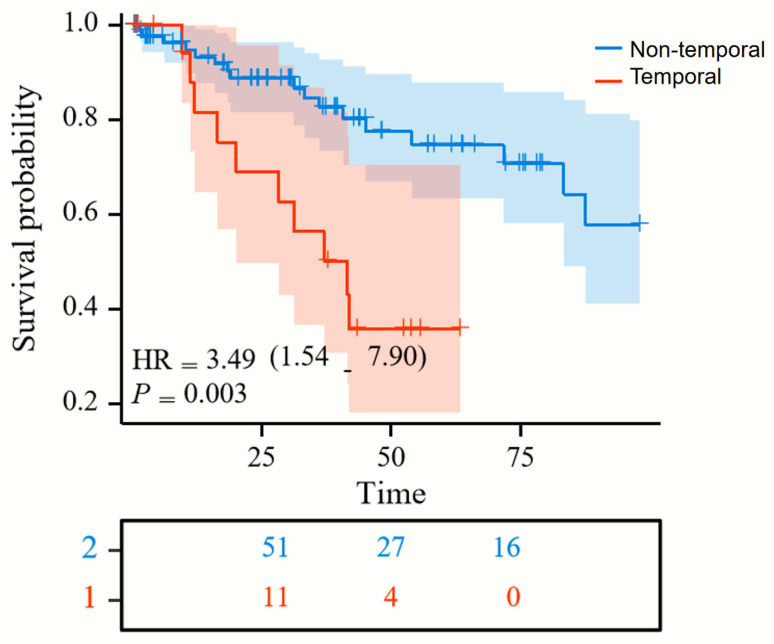
Kaplan–Meier overall survival curves for temporal versus non-temporal tumors in non-deep WHO grade 2–3 gliomas.

To further reduce anatomical heterogeneity, we performed an additional subgroup analysis restricted to non-deep WHO grade 2–3 gliomas. Within this subgroup, Kaplan–Meier analysis showed that patients with temporal tumors had significantly shorter OS than those with non-temporal tumors (log-rank *p* = 0.003, Figure 3).

This association was confirmed in Cox regression analysis. Compared with temporal tumors, non-temporal tumors showed a lower hazard of death in univariable analysis (HR 0.286, 95% CI 0.127–0.648, *p* = 0.003), and this association remained significant after adjustment for age, KPS, tumor size, preoperative epilepsy, radiotherapy, and Ki-67 (HR 0.275, 95% CI 0.113–0.666, *p* = 0.004). In the same model, higher KPS remained associated with better survival, and Ki-67 ≥ 30% remained independently associated with worse OS (Table 3).

These findings suggest that even within the non-deep subgroup, anatomical location retains prognostic relevance, with temporal involvement identifying a less favorable subset.

### 3.4. Prognostic Factors in WHO Grade 4 Gliomas

We next evaluated prognostic factors in WHO grade 4 gliomas. In univariable analysis, age, anatomical location, and radiotherapy were significantly associated with OS. Deep/midline tumors were associated with worse survival than non-deep tumors, and omission of radiotherapy was associated with a higher risk of death. Sex showed borderline significance, whereas KPS, tumor size, preoperative epilepsy, and Ki-67 were not significantly associated with OS.

In multivariable analysis, sex, anatomical location, and radiotherapy remained independently associated with survival. Female sex was associated with a lower hazard of death than male sex (HR 0.708, 95% CI 0.521–0.961, *p* = 0.027). Deep/midline location remained independently associated with worse OS (HR 2.196, 95% CI 1.408–3.425, *p* < 0.001). In addition, patients who did not receive radiotherapy had significantly worse survival than those who did (HR 2.325, 95% CI 1.636–3.304, *p* < 0.001). By contrast, Ki-67 did not show significant prognostic value in either univariable or multivariable analyses (Table 4).

**Figure 4:**
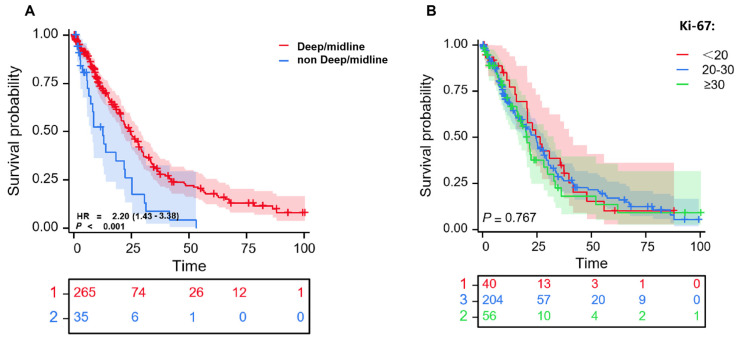
Overall survival according to location (**A**) and Ki-67 status (**B**) in WHO grade 4 gliomas.

Consistent with the Cox results, Kaplan–Meier analysis demonstrated significantly shorter OS in patients with deep/midline grade 4 tumors than in those with non-deep tumors, with early divergence of the survival curves (Figure 4).

### 3.5. Exploratory Analysis of Chemotherapy Duration in WHO Grade 4 Gliomas

After excluding cases with unavailable chemotherapy-duration data, we performed an exploratory analysis in 134 patients with WHO grade 4 gliomas to assess the association between chemotherapy duration and OS. Kaplan–Meier analysis showed significant survival differences across chemotherapy-duration groups (log-rank *p* = 0.047), with longer treatment duration generally associated with more favorable survival curves.

In univariable Cox analysis, longer chemotherapy duration was associated with improved OS (HR 0.943, 95% CI 0.895–0.993, *p* = 0.026). In multivariable analysis, this association was attenuated and approached, but did not reach, conventional statistical significance (HR 0.951, 95% CI 0.903–1.001, *p* = 0.057), whereas age remained significantly associated with OS (HR 1.021, 95% CI 1.000–1.041, *p* = 0.048).

**Figure 5:**
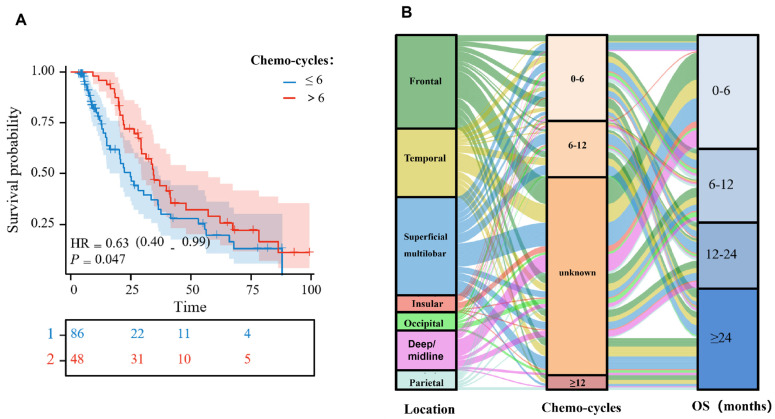
Association between chemotherapy duration and overall survival in WHO grade 4 gliomas. (**A**) Kaplan–Meier curves stratified by chemotherapy cycles (≤6 vs. >6). Shaded areas indicate 95% confidence intervals. (**B**) Sankey diagram showing the distribution of tumor location, chemotherapy cycles, and overall survival (OS in months).

Descriptive Sankey visualization further suggested that patients with longer chemotherapy duration were more frequently distributed in longer-survival categories. Taken together, these results indicate a possible association between treatment duration and survival in grade 4 gliomas, although this finding should be interpreted as exploratory (Figure 5).

### 3.6. Exploratory Molecularly Adjusted Analyses

To address the potential confounding effect of molecular classification, we performed additional exploratory Cox analyses restricted to cases with known IDH status and adjusted for IDH and MGMT status. In these analyses, deep/midline location remained independently associated with worse OS in both WHO grade 2–3 and grade 4 gliomas. By contrast, the prognostic effect of Ki-67 became less stable: it was no longer significant in grade 2–3 gliomas and showed an inconsistent pattern in grade 4 gliomas. These results are provided in Supplementary Tables S1 and S2 and support a cautious interpretation of Ki-67 as an exploratory marker.

### 3.7. Cross-Grade Comparison of Prognostic Patterns

To summarize the grade-dependent prognostic heterogeneity observed in our cohort, we compared the impact of classical clinicopathological factors across WHO grade 2–3 and grade 4 gliomas. Deep/midline tumor location consistently conferred an adverse prognosis in both grade groups. In contrast, Ki-67 appeared prognostically relevant in the primary grade 2–3 model, but its effect was attenuated after molecular adjustment and should therefore be interpreted cautiously, whereas treatment-related factors—including receipt of radiotherapy and chemotherapy duration—were more informative in grade 4 tumors. These findings highlight that the prognostic significance of conventional factors is context-dependent, and suggest that grade-specific interpretation may improve clinical risk stratification (Table 5).

## 4. Discussion

In this single-center retrospective cohort, we found that the prognostic relevance of classical clinicopathological factors was not uniform across WHO grade groups. Deep/midline location was consistently associated with worse overall survival in both grade 2–3 and grade 4 gliomas, whereas Ki-67 showed prognostic relevance in the primary grade 2–3 analysis but became less stable after molecular adjustment, whereas treatment-related variables appeared more informative in grade 4 disease. And treatment-related variables appeared more informative in grade 4 disease. These findings support the view that conventional prognostic factors in glioma should be interpreted within a grade-specific clinical context rather than assumed to have stable meaning across all glioma entities.

One of the strongest findings in our cohort was the adverse prognostic effect of deep/midline location. This is broadly consistent with prior studies showing that tumor location carries prognostic information in glioblastoma and diffuse glioma beyond simple descriptive anatomy. In our additional exploratory analyses restricted to cases with known IDH status and adjusted for IDH and MGMT, deep/midline location remained an independent adverse prognostic factor in both grade 2–3 and grade 4 gliomas, supporting the relative robustness of this association after partial molecular adjustment (Supplementary Tables S1 and S2). A recent multicenter Neuro-Oncology Advances study reported that clinical variables, tumor size, and location all contributed to overall survival prediction in glioblastoma [14]. Statistical atlas–based work has further shown that centrally located tumors, particularly those involving the corpus callosum or basal ganglia, are enriched among patients with the shortest survival [10]. In parallel, a systematic review of gliomas infiltrating the corpus callosum emphasized the substantial surgical difficulty and poor outcomes associated with these tumors [15]. These findings support the clinical relevance of deep/midline anatomy as a prognostic marker.

However, this association should be interpreted cautiously. Because postoperative extent of resection (EOR) and residual tumor volume were unavailable, we cannot determine whether the observed effect mainly reflects limited maximal safe resection in eloquent deep structures or intrinsic biological aggressiveness. In glioblastoma, greater EOR and lower residual tumor burden are consistently associated with improved survival [16,17]. Moreover, glioblastomas with deep supratentorial extension have lower rates of gross total resection and worse survival, with the adverse effect appearing largely related to surgical limitations [18]. Adult thalamic glioma studies also suggest that complete or maximal safe resection, when feasible in selected patients, is associated with longer survival [19].

Conversely, a deep/midline location may also mark high-risk biology. Diffuse midline glioma, H3 K27-altered, is classified as a CNS WHO grade 4 molecular entity [3], and H3 K27M/H3 K27-altered diffuse midline gliomas show poor outcomes that may be independent of histological grade, specific midline site, or EOR [20,21]. In addition, tumors involving central white matter pathways may exhibit broader network-level infiltration, and connectome-based studies have linked structural connectivity patterns with survival in glioblastoma [22,23]. Therefore, deep/midline location is best interpreted as a composite phenotypic marker reflecting both reduced surgical accessibility and unfavorable biological context [24,25]. Future studies incorporating quantitative EOR, postoperative residual tumor volume, molecular classification, and tractography/connectome features are needed to distinguish the direct effect of location from effects mediated by resectability and tumor biology.

Within the non-deep WHO grade 2–3 subgroup, we further observed worse survival for temporal tumors. This finding should be interpreted cautiously, but it is not without precedent. Prior imaging-based studies in glioblastoma have reported that some temporal subregions, particularly central or left temporal areas, may be linked to worse survival, although the spatial pattern is heterogeneous across cohorts and mapping methods [4,10]. Our result is therefore directionally compatible with a broader literature suggesting that not all superficial or lobar tumors behave similarly. However, unlike the deep/midline finding, the temporal result should be regarded as hypothesis-generating rather than definitive [26]. It may reflect differences in surgical corridor complexity, language-network constraints, mesial temporal or insular extension, or unmeasured biological features that covary with location. This is also an area where future analyses integrating molecular subtype and extent of resection would be especially valuable.

Ki-67 showed a different, grade-dependent pattern. In our cohort, higher Ki-67 was independently associated with worse survival in grade 2–3 gliomas, whereas no clear independent prognostic effect was observed in grade 4 tumors. This pattern is compatible with previous literature showing that Ki-67 has overall prognostic value in glioma but with substantial heterogeneity across tumor groups [12,27]. After additional adjustment for IDH and MGMT in cases with known IDH status, the prognostic effect of Ki-67 became unstable. It was no longer independently associated with OS in grade 2–3 gliomas and showed an inconsistent pattern in grade 4 tumors, indicating that its prognostic value should be interpreted cautiously (Supplementary Tables S1 and S2). A meta-analysis concluded that higher Ki-67 is generally associated with poorer outcomes in gliomas, while also emphasizing between-study heterogeneity. More specifically, work in lower-grade diffuse gliomas has suggested that Ki-67 can refine risk stratification and may be most informative when interpreted alongside molecular characteristics rather than histology alone [28].

Several prior studies support the idea that Ki-67 may be particularly relevant in lower-grade disease. In lower-grade glioma, radiomic studies have linked Ki-67 expression to both imaging phenotype and survival, implying that proliferative activity still carries discriminatory value in tumors with more heterogeneous clinical trajectories [29]. Likewise, studies integrating Ki-67 with molecular markers in lower-grade glioma have argued that proliferation markers retain utility because they capture biological aggressiveness not fully explained by grade alone [4]. Our findings are broadly compatible with this view in the primary analysis; however, the attenuation of Ki-67 after IDH/MGMT adjustment suggests that its prognostic value in grade 2–3 gliomas may partly reflect molecular context rather than a fully independent effect.

By contrast, the literature in glioblastoma is notably less consistent, which helps explain why we did not observe a significant prognostic effect of Ki-67 in grade 4 disease. A population-based study using digital pathology and exclusion of non-neoplastic proliferating cells found that Ki-67 labeling index did not provide strong independent prognostic information once relevant covariates were considered [30]. Similar uncertainty appears in other glioblastoma cohorts, where Ki-67 sometimes shows association in univariable analysis but loses significance after multivariable adjustment [13]. Taken together, these data support a cautious interpretation: Ki-67 may remain biologically relevant in grade 4 glioma, but its standalone clinical prognostic utility appears weaker and less reproducible than in lower-grade tumors.

Treatment-related variables showed the opposite pattern from Ki-67, appearing more informative in grade 4 gliomas. In our study, receipt of radiotherapy was independently associated with better survival, and longer chemotherapy duration showed a favorable trend in exploratory analysis. This is directionally consistent with the broader glioblastoma literature, where survival is strongly influenced by whether patients can receive and complete standard multimodal therapy. Real-world outcome studies continue to show that treatment eligibility and delivery are major determinants of survival in glioblastoma cohorts [14]. In addition, recent work has shown that prolonged overall treatment time and radiotherapy interruptions are independently associated with inferior survival in IDH-wildtype glioblastoma, reinforcing the clinical importance of treatment completion and continuity [31].

The chemotherapy-duration finding deserves especially cautious discussion. Extended adjuvant temozolomide has been investigated repeatedly, but the literature remains mixed. A recent NOA systematic review and meta-analysis reported that extended adjuvant TMZ was associated with improved progression-free and overall survival, but the pooled signal was driven largely by nonrandomized comparative studies [32], while randomized evidence remained less definitive [33]. More recent retrospective series have also reported potential benefit from prolonged TMZ, yet these studies are vulnerable to the same core bias: patients who tolerate treatment longer often already have better baseline condition, slower disease progression, or more favorable molecular biology [34,35]. For that reason, our result should be framed as exploratory and hypothesis-generating rather than causal evidence that extending chemotherapy independently improves survival.

An additional reason our treatment-related findings may appear more prominent in grade 4 disease is that the outcome in glioblastoma is often compressed into a relatively short time window, making differences in treatment access, adherence, interruptions, and cumulative exposure more visible. In contrast, lower-grade gliomas often have more prolonged and heterogeneous natural histories, in which anatomical context and intrinsic proliferative behavior may retain stronger relative prognostic value [36]. This interpretation is also consistent with contemporary WHO 2021–era survival analyses emphasizing that clinical outcomes in glioma emerge from the interaction of molecular subtype, treatment delivery, and conventional clinicopathological factors rather than from any single variable alone [37].

Several limitations should be acknowledged. First, this was a retrospective single-center study with potential selection bias and limited generalizability, and follow-up duration may be insufficient for evaluating long-term prognostic stability in lower-grade gliomas. Second, molecular annotation was incomplete relative to current WHO 2021 standards; although exploratory adjustment for IDH and MGMT was performed, residual molecular confounding cannot be excluded. Third, the extent of resection and residual tumor volume were unavailable, so the adverse association of deep/midline location with survival should be viewed as a robust clinical observation rather than evidence of location-specific biology alone. Finally, some subgroup analyses were limited by small sample size, and multiple exploratory comparisons were not adjusted for multiplicity.

In conclusion, our findings support a grade-specific framework for prognostic assessment in glioma. Deep/midline location appears to be a relatively stable adverse prognostic factor across grade groups, whereas Ki-67 is more informative in grade 2–3 gliomas and treatment-related variables appear more clinically informative in grade 4 disease. Future multicenter studies with complete molecular annotation, surgical variables, and treatment-course detail will be needed to validate and refine these observations.

## 5. Conclusions

In this single-center retrospective study of 429 patients with glioma, we demonstrated that the prognostic significance of classical clinicopathological factors varies across WHO grades. Deep/midline tumor location was consistently associated with worse overall survival in both grade 2–3 and grade 4 gliomas and remained relatively robust after partial molecular adjustment. In contrast, Ki-67 showed prognostic relevance mainly in the primary grade 2–3 analysis but became less stable after adjustment for IDH and MGMT, supporting cautious interpretation as an exploratory, molecular-context-dependent marker. Treatment-related variables, including radiotherapy and chemotherapy duration, appeared more informative in grade 4 gliomas. These findings support a grade-specific and molecular context-aware interpretation of conventional prognostic factors. Future multicenter studies incorporating comprehensive molecular annotation, standardized surgical variables, and detailed treatment-course data are warranted to validate and refine these observations.

## Figures and Tables

**Table 1:** Comparison of clinical, pathological, and molecular characteristics among patients with WHO grade 2, grade 3, and grade 4 gliomas.

Variable	WHO Grade 2	WHO Grade 3	WHO Grade 4	*p*
Gender				0.484
Female	19 (38.0%)	34 (43.0%)	140 (46.7%)	
Male	31 (62.0%)	45 (57.0%)	160 (53.3%)	
Age				0.000
median (IQR)	45.0 (34.0–50.0)	48.0 (38.0–58.0)	55.0 (48.0–65.0)	
KPS	90.0 (90.0–90.0)	90.0 (90.0–90.0)	90.0 (80.0–90.0)	0.018
Ki67				0.000
median (IQR)	5.0 (3.2–9.5)	15.0 (10.0–30.0)	30.0 (20.0–40.0)	
Tumor diameter	5.0 (4.0–5.0)	5.0 (4.0–6.0)	5.0 (4.0–6.0)	0.063
epilepsy				0.000
no	26 (52.0%)	61 (77.2%)	263 (87.7%)	
yes	24 (48.0%)	18 (22.8%)	37 (12.3%)	
Location				0.262
Deep/midline	2 (4.0%)	9 (11.4%)	35 (11.7%)	
Non-deep/midline	48 (96.0%)	70 (88.6%)	265 (88.3%)	
Lobe				0.000
Superficial multilobar	4 (8.0%)	11 (13.9%)	80 (26.7%)	
Insular	7 (14.0%)	12 (15.2%)	15 (5.0%)	
Occipital	0 (0.0%)	0 (0.0%)	15 (5.0%)	
Deep/midline	2 (4.0%)	9 (11.4%)	35 (11.7%)	
Parietal	2 (4.0%)	3 (3.8%)	15 (5.0%)	
Frontal	28 (56.0%)	31 (39.2%)	82 (27.3%)	
Temporal	7 (14.0%)	13 (16.5%)	58 (19.3%)	
Pathology				0.000
NOS	0 (0.0%)	3 (3.8%)	3 (1.0%)	
Oligodendroglioma	17 (34.0%)	40 (50.6%)	0 (0.0%)	
Astrocytoma	33 (66.0%)	36 (45.6%)	10 (3.3%)	
Glioblastoma	0 (0.0%)	0 (0%)	287 (95.7%)	
Radiotherapy				0.190
no	25 (50.0%)	27 (34.2%)	116 (38.7%)	
yes	25 (50.0%)	52 (65.8%)	184 (61.3%)	
Chemotherapy				0.031
no	29 (58.0%)	28 (35.4%)	121 (40.3%)	
yes	21 (42.0%)	51 (64.6%)	179 (59.7%)	
IDH				0.000
unknown	11 (22.0%)	20 (25.3%)	147 (49.0%)	
No mutation	6 (12.0%)	20 (25.3%)	135 (45.0%)	
mutation	33 (66.0%)	39 (49.4%)	18 (6.0%)	
MGMT methyl				0.000
No-methylation	2 (4.0%)	8 (10.1%)	55 (18.3%)	
unknown	26 (52.0%)	29 (36.7%)	184 (61.3%)	
methylation	22 (44.0%)	42 (53.2%)	61 (20.3%)	
TERT				0.002
C250T mutation	2 (4.0%)	8 (10.1%)	22 (7.3%)	
C288Tmutation	10 (20.0%)	20 (25.3%)	72 (24.0%)	
unknown	18 (36.0%)	27 (34.2%)	156 (52.0%)	
No mutation	20 (40.0%)	24 (30.4%)	50 (16.7%)	
P53 expression				0.097
+	33 (66.0%)	49 (62.0%)	221 (73.7%)	
−	17 (34.0%)	30 (38.0%)	79 (26.3%)	
1p19q				0.000
co-deletion	11 (22.0%)	30 (38.0%)	0 (0)	
No co-deletion	24 (48.0%)	31 (39.2%)	195 (65.0%)	
unknown	15 (30.0%)	18 (22.8%)	105 (35.0%)	

**Table 2:** Univariate and multivariate Cox regression analyses of prognostic factors for OS in patients with WHO grade 2–3 gliomas.

Characteristic	Total No.	Univariate Analysis		Multivariate Analysis	
		HR (95% CI)	*p* Value	HR (95% CI)	*p* Value
Gender	129				
Male	76	Reference			
Female	53	0.742 (0.372–1.479)	0.396		
Age	129	1.026 (0.997–1.055)	0.074	1.005 (0.977–1.034)	0.723
KPS	129	0.977 (0.960–0.995)	**0.012**	0.971 (0.952–0.991)	**0.005**
Location	129				
Non-Deep/midline	118	Reference		Reference	
Deep/midline	11	5.494 (2.466–12.238)	**<0.001**	4.939 (1.997–12.215)	**<0.001**
Tumor diameter	129	0.906 (0.747–1.098)	0.313		
Epilepsy	129				
yes	42	Reference			
no	87	1.721 (0.825–3.589)	0.148		
Radiotherapy	129				
no	52	Reference			
Yes	77	0.779 (0.383–1.585)	0.490		
Chemo-Cycle	58	1.048 (0.962–1.143)	0.283		
Ki-67	129				
<20	89	Reference		Reference	
20~30	16	1.480 (0.500–4.385)	0.479	1.135 (0.352–3.658)	0.832
≥30	24	2.667 (1.335–5.328)	**0.005**	2.194 (1.064–4.526)	**0.033**

Proportional hazards assumption was verified by Schoenfeld residuals (global *p* = 0.382). Bold values indicate statistical significance at *p* < 0.05.

**Table 3:** Cox regression analyses of the association between temporal location and overall survival in non-deep WHO grade 2–3 gliomas.

Characteristic	Total No.	Univariate Analysis		Multivariate Analysis	
		HR (95% CI)	*p* Value	HR (95% CI)	*p* Value
Age	118	1.040 (1.007–1.075)	**0.018**	1.021 (0.988–1.056)	0.210
KPS	118	0.971 (0.953–0.989)	**0.002**	0.969 (0.949–0.989)	**0.003**
**Location**	118				
Temporal	20	Reference		Reference	
Non-temporal	98	0.286 (0.127–0.648)	**0.003**	0.275 (0.113–0.666)	**0.004**
**Ki-67**	118				
<20%	83	Reference		Reference	
20–30%	16	1.866 (0.612–5.688)	0.273	1.514 (0.471–4.873)	0.487
≥30%	19	2.677 (1.203–5.959)	**0.016**	3.314 (1.424–7.716)	**0.005**

Multivariable analysis was adjusted for age, KPS, and Ki-67. Bold values indicate statistical significance at *p* < 0.05.

**Table 4:** Univariate and multivariate Cox regression analyses of prognostic factors for OS in patients with WHO grade 4 gliomas.

Characteristic	Total No.	Univariate Analysis		Multivariate Analysis	
		HR (95% CI)	*p* Value	HR (95% CI)	*p* Value
Gender	300				
Male	160	Reference		Reference	
Female	140	0.739 (0.545–1.001)	0.051	0.708 (0.521–0.961)	**0.027**
Age	300	1.016 (1.003–1.028)	**0.012**	1.007 (0.995–1.020)	0.270
**Location**	300				
Non-Deep/midline	265	Reference		Reference	
Deep/midline	35	2.196 (1.428–3.377)	**<0.001**	2.196 (1.408–3.425)	**<0.001**
KPS	300	0.991 (0.982–1.001)	0.066	1.004 (0.994–1.014)	0.442
Tumor diameter	300	1.000 (0.997–1.003)	0.934		
Epilepsy	300				
no	263	Reference			
yes	37	0.697 (0.403–1.207)	0.198		
Radiotherapy	300				
yes	184	Reference		Reference	
no	116	2.350 (1.718–3.216)	**<0.001**	2.325 (1.636–3.304)	**<0.001**
**Ki-67**	300				
<20	40	Reference			
≥30	204	1.084 (0.692–1.699)	0.723		
20~30	56	1.213 (0.710–2.071)	0.480		

Proportional hazards assumption was verified by Schoenfeld residuals (global *p* = 0.163). Bold values indicate statistical significance at *p* < 0.05.

**Table 5:** Cross-grade comparison of prognostic patterns of classical clinicopathological factors in gliomas.

Factor	Grade 2–3	Grade 4	Interpretation
**Deep/midline location**	Significant	Significant	Relatively stable adverse factor across grades
**Ki-67**	Significant in the primary model; attenuated after molecular adjustment	Not consistently significant	Exploratory and molecular-context dependent
**Chemo cycles**	Weak/not clear	More relevant	Treatment-related impact more visible in grade 4

## Data Availability

The original contributions presented in this study are included in the article/Supplementary Material. Further inquiries can be directed to the corresponding authors.

## Supplementary Materials

The following supporting information can be downloaded at: https://www.mdpi.com/article/10.3390/jcm15114372/s1, Table S1. Cox regression analyses for prognostic factors of WHO grade 2–3 gliomas after excluding patients with unknown IDH status; Table S2. Cox regression analyses for prognostic factors of WHO grade 4 gliomas after excluding patients with unknown IDH status.
