# Supplementary material for: Grade-Dependent Prognostic Value of Classical Clinicopathological Factors in Glioma: A Single-Center Retrospective Study
Source: J Clin Med. 2026 Jun 5;15(11):4372. doi: 10.3390/jcm15114372 (PMC13257477; doi:10.3390/jcm15114372)
Supplement: Supplementary file 1 [file jcm-15-04372-s001.zip › jcm-4310932-supplementary.pdf]

Supplementary Table S1. Cox regression analyses for prognostic factors of WHO grade 2–3 gliomas after excluding patients with unknown IDH status

Characteristics	Total(N)	Univariate analysis		Multivariate analysis	
		Hazard ratio (95% CI)	P value	Hazard ratio (95% CI)	P value
Age	98	1.053 (1.008 - 1.100)	**0.020**	1.013 (0.974 - 1.054)	0.523
Tumor location	98				
Non-deep	89	Reference		Reference	
**Deep/ midline**	9	6.057 (2.268 - 16.181)	**< 0.001**	4.178 (1.281 - 13.619)	**0.018**
KPS	98	0.975 (0.956 - 0.995)	**0.014**	0.973 (0.950 - 0.995)	**0.019**
**IDH status**	98				
Mutant	72	Reference		Reference	
Wildtype	26	5.796 (2.167 - 15.503)	**< 0.001**	3.265 (1.054 - 10.111)	**0.040**
MGMT methyl	98				
Methylated	64	Reference			
Unknown	24	0.816 (0.299 - 2.229)	0.691		
Unmethylated	10	0.711 (0.156 - 3.248)	0.660		
Ki67 group	98				
<20	77	Reference			
20~30	9	1.218 (0.158 - 9.417)	0.850		
≥30	12	2.367 (0.836 - 6.701)	0.105		

Proportional hazards assumption was verified by Schoenfeld residuals (global p = 0.302).

Supplementary Table S2. Cox regression analyses for prognostic factors of WHO grade 4 gliomas after excluding patients with unknown IDH status

Characteristics	Total(N)	Univariate analysis		Multivariate analysis	
		Hazard ratio (95% CI)	P value	Hazard ratio (95% CI)	P value
Age	153	1.023 (1.003 - 1.043)	**0.025**	1.014 (0.994 - 1.035)	0.162
Tumor location	153				
Non-deep	136	Reference		Reference	
**Deep/ midline**	17	2.385 (1.306 - 4.357)	**0.005**	2.604 (1.355 - 5.002)	**0.004**
KPS	153	0.989 (0.978 - 1.000)	0.059	0.991 (0.979 - 1.003)	0.132
**IDH status**	153				
Wildtype	135	Reference		Reference	
Mutant	18	0.408 (0.184 - 0.908)	**0.028**	0.641 (0.272 - 1.508)	0.308
MGMT methyl	153				
Methylated	59	Reference		Reference	
Unmethylated	53	1.649 (0.948 - 2.869)	0.077	1.876 (1.064 - 3.306)	**0.030**
Unknown	41	1.820 (1.046 - 3.166)	**0.034**	1.846 (1.016 - 3.353)	**0.044**
Ki67_group2	153				
<20	19	Reference		Reference	
≥30	101	1.337 (0.632 - 2.828)	0.447	1.894 (0.862 - 4.163)	0.112
20~30	33	2.289 (0.998 - 5.249)	0.050	2.970 (1.220 - 7.232)	**0.016**

Proportional hazards assumption was verified by Schoenfeld residuals (global p = 0.944).
